# Building in-house PBPK modelling tools for oral drug administration from literature information

**DOI:** 10.5599/admet.638

**Published:** 2019-02-23

**Authors:** Silvia Grandoni, Nicola Cesari, Giandomenico Brogin, Paola Puccini, Paolo Magni

**Affiliations:** 1 Università degli Studi di Pavia, Dept. Electrical, Computer and Biomedical Engineering, Pavia, Italy; 2 Chiesi Farmaceutici S.p.A, Pharmacokinetic, Biochemistry and Metabolism Department, Parma, Italy

**Keywords:** Model Based Drug Development, PBPK models, Oral administration, Pharmacokinetics, Model-based prediction, In Vitro In Vivo Extrapolation

## Abstract

The interest in using physiologically-based pharmacokinetic (PBPK) models as a support to the drug development decision making process has rapidly increased in the last years. These kind of models are examples of the “bottom up” modelling strategy, which progressively integrates into a mechanistic framework different information as soon as they become available along the drug development. For this reason PBPK models can be used with different aims, from the early stages of drug development up to the clinical phases. Different software tools are nowadays available. They can be categorized in “designed software” and “open software”. The first ones typically include commercial platforms expressly designed to implement PBPK models, in which the model structure is pre-defined, assumptions are generally not explicitly declared and equations are hidden to the user. Even if the software is validated and routinely used in the pharmaceutical industry, sometimes they do not allow working with the flexibility needed to cope with specific applications/tasks. For this reason, some scientists prefer to define and implement their own PBPK tool in “open” software. This paper shows how to build an in-house PBPK tool from species-related physiological information available in the literature and a limited number of drug specific parameters generally made available by the drug development process. It also reports the results of an evaluation exercise that compares simulated plasma concentration-time profiles and related pharmacokinetic (PK) parameters (i.e., AUC, C_max_ and T_max_) with literature and in-house data. This evaluation involved 25 drugs with different physico-chemical properties, intravenously or orally administrated in three different species (i.e., rat, dog and man). The comparison shows that model predictions have a good degree of accuracy, since the average fold error for all the considered PK parameters is close to 1 and only in few cases the fold error is greater than 2. In summary, the paper demonstrates that addressing specific aims when needed is possible by creation of in-house PBPK tools with satisfactory performances and it provides some suggestions how to do that.

## Introduction

Drug development is an extremely long and expensive process. Pharmaceutical companies look for methodologies able to reduce the attrition rate and to speed-up the whole process. The *in silico* study of the pharmacokinetics (PK) through modelling and simulation (M&S) can help on this and, in the last decades, received attention of both industries and regulatory authorities [1,2]. One of the possible strategies that allows the *in silico* study of ADMET is the use of physiologically-based pharmacokinetic (PBPK) models.

In PBPK models the living organism is described as a set of compartments representing organs and tissues connected by the blood circulation. Hence, differently from the compartmental modelling approach, in PBPK models there is a direct correspondence between anatomical structures and model compartments. The time course of the drug concentration in each compartment is described through a mass-balance equation [3]. Specific reactions if needed and known can be easily added in the different compartments, providing a more detailed mechanistic description. One of the most interesting characteristics of PBPK models is that, as in systems pharmacology, model parameters can be divided into system-specific (e.g., organ volumes and blood fluxes) and compound-related (e.g., solubility, permeability, tissue binding). This means that it is possible to create pre-built drug-independent model structures that can be re-used for predicting the exposure to different compounds or to xenobiotics in species different from those for which the model was originally developed [3,4]. In fact, one of the first and more extensive field of PBPK modelling approach application was the preclinical to clinical translation in toxicology, where for ethical reasons, the exposure of humans to pollutants or toxic agents is not allowed and then it is only possible to predict the human exposure on the basis of preclinical data [5].

In the literature, examples of application and assessment of PBPK models of different complexity are reported along the whole drug development process. Different type of information can be integrated from the beginning in the PBPK mechanistic framework and gradually added, updated and refined as they are made available by the *in vitro*/*in vivo* studies. Therefore, PBPK modelling approach represents a good example of incremental strategy to the model building.

One possible application of PBPK models in early stages of drug development is to prioritize candidates before performing *in vivo* experiments. The cost to generate *in vivo* data is in fact higher than that to characterize candidates *in vitro* (e.g., their main properties can be routinely determined via high-throughput techniques). The *in vitro* candidate properties can hence be combined with physiological species-specific information to predict the *in vivo* PK in the preclinical species of interest [6-8]. Once preclinical *in vivo* data become available for the selected candidate, the simulated profiles can be compared with experimental data to verify if some of the model assumptions were violated. If this is the case, the model can be refined following the “learn, confirm and refine” paradigm. The adjusted model can be further used and expanded, for example, for predicting other routes of administration (e.g., oral - PO - after intravenous - IV) in the same species or the same route in other species. For example, PBPK models can be used to extrapolate PK from animals to man (e.g., healthy volunteers), by updating the species-specific model parameters and collecting additional compound-related *in vitro* data [8,9]. Furthermore, within the clinical development context, PBPK models can be used to extrapolate PK from healthy volunteers to patients or can be used to predict PK in special populations, like children and elderly, integrating into the model the physiological-related changes due to the specific physiological or pathological condition [2].

In recent years, the number of PBPK model publications and regulatory submissions has grown rapidly and the regulatory agencies are working to define the best practice on development, qualification, application and reporting of PBPK modelling activities [10]. In 2016, EMA published a draft version of a guideline about that and, in 2018, FDA provided guidelines about PBPK analysis formats and contents of applications [11,12]. Furthermore, a number of drug labels are informed by the results of PBPK model simulations as documented by Jamei [4]. This huge increase in the use of PBPK models has been made possible by factors such as the increased computational power necessary to handle mathematical complexity, the improvement in biological systems understanding and the advancing in drug property predictions from *in vitro* and *in silico* data [4,8].

Nowadays, scientists have in their hands two kind of tools for working with PBPK models: the “designed” software and the “open” software [1]. The first group refers mainly to commercial software platforms, in which generally the PBPK model structure is pre-defined, assumptions are not completely made explicit and equations are generally hidden to the user. They also include often ready to use databases of parameter values and population libraries that allow, for example, simulation of PK profiles in different conditions and for different demographics. Examples of such software are GastroPlus™ (Simulation Plus Inc.), PK-sim (Bayer Technologies Services), Simcyp Simulator™ (Certara). The second group refers to software not specifically designed to work with PBPK models, but originally developed for engineers, mathematicians and statisticians, in which the user needs to write the code with the equations to implement the model from scratch. Examples of “open” software are MATLAB (The Mathworks Inc.), R (R Development Core Team), Berkeley-Madonna (University of California), SAAM II (University of Washington).

“Designed” software helped the diffusion of PBPK models for their intrinsic advantages such as the presence of user-friendly interfaces, the fact that the user does not need to have expertise in programming languages, the specific scientific-technical support by software vendors and, mainly, the huge amount of knowledge encoded in these tools. For the same reasons, the main risk in using these software tools is that, if the user has not sufficient awareness about the model assumptions and the parameter relationships, the results can be misinterpreted and due to the high complexity of the encoded models can be very difficult to understand which parameters or processes are responsible of possible poor predictions. With the “open software” the model is directly implemented by the user, hence, a deep understanding of the model assumptions, interdisciplinary skills ranging from physiology to programming and enough modelling expertise are needed. Model parameter values have to be found somewhere directly by the modeller. This is not an easy task and necessarily leads to build simpler models, but perfectly adequate to address at least some of the questions. One advantage of an open model structure is that it can be personalized to cope with specific problems such as the study of a particular route of administration or the study of the pharmacodynamics (PD), through a PBPK-PD model [1]. Not only the academia but also companies are using open software for PBPK modelling. Some of them use in-house developed tools, tailored on specific needs, to assist their drug development process [13].

Basic PBPK models for IV administrations can be built from literature information. Then, they can be extended by integrating other routes of administration to study, for example gastrointestinal absorption (examples implemented in open software are reported in [13]) or special routes such as pulmonary [14] or vaginal delivery [15], or more generally, by integrating specific ADME processes. In this work, through an example, it is shown how this process can be actually done. A basic PBPK model for IV administrations in different species (rat, dog, man) is built and then extended to PO administrations. To help the reader, all the relevant knowledge collected form the literature, such as parameter values or mathematical relationships, is summarized in the Supplementary material. The proposed model has been implemented in MATLAB and evaluated simulating IV and PO administration experiments in three species (rat, dog, man) for different compounds and comparing simulated profiles with published and in-house data.

**Figure 1.**
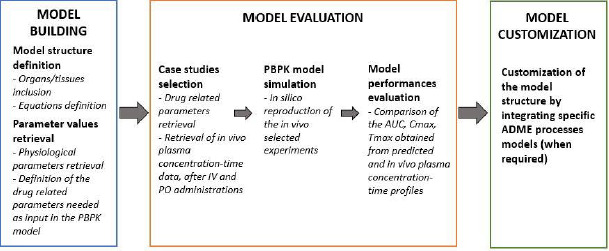
A schematic representation of the proposed workflow for model building, evaluation and application.

Figure 1 summarizes the key points of the workflow suggested in the paper and presented in detail in the next sections, related to model building, model evaluation and model customization to cope with specific case studies.

## Model building

### Model structure: the compartments

**Figure 2.**
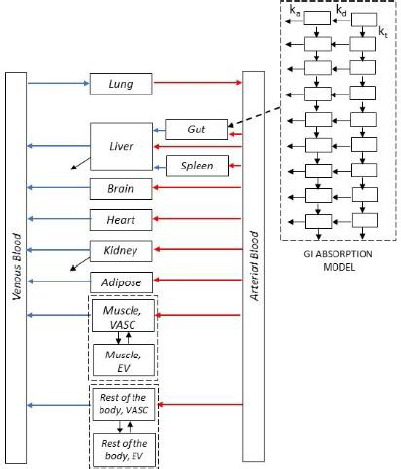
A basic IV PBPK model structure extended with a gastrointestinal absorption model.

PBPK models consist of a series of compartments representing the body organs/tissues. Those typically included in a basic PBPK model are: adipose, brain, gut, heart, kidneys, lung, muscle, skin and spleen [3,7,16]. Depending on the specific needs, other tissues can be included or lumped together. Fig. 2 shows the basic structure adopted in this work.

The drug concentration *C*_T_ in the generic tissue T is governed by the following mass balance differential equation:


(1)

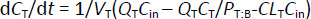



where *V*_T_ and *Q*_T_ are the tissue volume and blood flux, respectively; *C*_in_ is the blood drug concentration in input to the tissue, that is the arterial blood concentration for all the tissues except for the lung which receives the venous blood; *P*_T:B_ is the tissue to blood partition coefficient; *CL*_T_ is the tissue clearance, present in some of the tissues [7].

The equation for the venous compartment, receiving as input the output of all the other tissues (except lung) has the following expression:


(2)

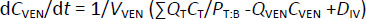



where *C*_VEN_, *V*_VEN_ and *Q*_VEN_ are the venous drug concentration, volume and flux, respectively, and *D*_IV_ represents the IV dose, if present. The mass balance equation for the arterial compartment can be analogously derived as:


(3)

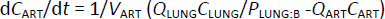



where *C*_ART_, *V*_ART_ and *Q*_ART_ are the arterial drug concentration, volume and flux, respectively. Fluxes *Q*_VEN_, *Q*_ART_ and *Q*_LUNG_ are equal to the cardiac output.

Each tissue could be modelled as perfusion-limited or permeability-limited. The first situation generally occurs for small lipophilic compounds for which the tissue perfusion is the step limiting the absorption and it is described through the Eq. (1). Instead, the second situation generally occurs for large hydrophilic compounds for which the permeation through the cell membrane is the limiting step. To model that, the tissue compartment can be divided in two parts: the extravascular space and the vascular space, divided by a membrane which acts as a barrier [3]. Then, typical equations of a permeability-limited tissue (without clearance) can be the following ones:


(4)

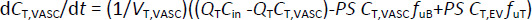




(5)

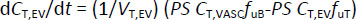



where *C*_T,VASC_ is the drug concentration in the vascular part of the tissue T and *V*_T,VASC_ its volume; *C*_T,EV_ and *V*_T,EV_ are the corresponding quantities for the extravascular part of the tissue; *PS* is the permeability-surface product which takes into account the limitation of the permeability that prevents a fast distribution of the drug into the tissue; *f*_uB_ and *f*_uT_ are the unbounded drug fraction in blood and tissue, respectively [17]. They can be computed as *f*_uB_= *f*_uP_/*BP* and *f*_uT_= *f*_uB_/*P*_T:B_, where *f*_uP_ is the unbounded drug fraction in plasma and BP is blood to plasma ratio.

To describe PO administrations, a model of gastrointestinal absorption has to be added to the basic structure just described, which is able to predict tissue drug concentrations only after an IV administration. In this paper, a simplified version of the advanced compartmental absorption and transit (ACAT) model was adopted [18]. The gastrointestinal system was divided in 9 compartments, the first, receiving the solid drug, is the stomach, the other segments represent the small intestine and the last compartment the colon. Each intestinal section is composed by a compartment in which the drug is in its undissolved form (from here it can dissolve or transit in the same form in the subsequent section) and by a compartment in which the drug is dissolved in the physiological fluids (from there drug can be absorbed or transit in the subsequent section; in colon drug can also be excreted). Two detailed examples of PBPK models in which the gastrointestinal absorption modelling is based on the ACAT model can be found in [16,19].

As discussed in the introduction, in PBPK models it is possible to distinguish from system-specific and compound-related parameters. In the next subsections, it will be discussed which are the parameters that can be found in the literature and which, instead, need to be derived from *in vitro* experiments through *in vitro-in vivo* extrapolation techniques [20].

### System-specific parameters

System-specific parameters, such as blood fluxes and volumes, can be found in the literature; some of the sources frequently used in PBPK modelling are [21,22], in which values are reported for different species. Values used to build the here proposed PBPK model were taken for all the tissues from them. The volume of blood was taken from [22]. It was divided in arterial and venous blood following [22], in which a 75 % *vs* 25 % was suggested for arterial and venous blood volume, respectively. The volume of the rest of the body compartment was calculated assuming that all the tissues included in the model represent the 85 % of the total body weight (BW) [23]. Hence, its weight is calculated subtracting the sum of the weights of all the other compartments from the 85 % of BW.

The volumes of the extravascular and vascular compartments for the tissues with a permeability-limited kinetics can be calculated from the fractions of vascular and interstitial spaces, that can be found for rats in [24]. In the model here proposed, the vascular compartment includes also the interstitial space. For other species, it was assumed that that the ratio between extravascular and vascular volumes is the same as in rats [25]. Organ surfaces needed to compute the permeability-surface product (*PS*) for permeability limited organs (muscle and rest of the body in model of Figure 2) according to the authors’ knowledge are not present in the literature, except for lung for which volumes and surfaces for the tracheobronchial and alveolar regions are available [4]. Hence, in this work, organ surfaces for other tissues were obtained by scaling the surface area from the tracheobronchial region through the following formula:


(6)

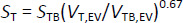



where *S*_T_ and *V*_T,EV_ are the surface and the extravascular volume of the tissue of interest, respectively; analogously, *S*_TB_ and *V*_TB,EV_ are the tracheobronchial surface and extravascular volume, respectively.

For what concerns the parametrization of the gastrointestinal absorption model, lumen volumes of the different intestinal segments and the relative pH values were obtained from [25] for rat, dog and man. The transit constants *K*_t_ can be calculated from the mean residence time (MRT) as 1/MRT. MRTs of small intestine segments can be obtained dividing the total small intestine MRT reported in the literature by 7 (assuming, hence, that the transit time is the same for each segment). MRT values are summarized in Table 1 with the respective sources. Intestinal radius values for all the species can be found in [25].

All the parameters values discussed in this section are reported for the three considered species in the Supplementary Material (*System-specific parameter values* section).

### Compound-related parameters

In case of PO drug delivery, before the drug is absorbed, it undergoes to the dissolution process. The dissolution process depends on the physico-chemical characteristics of the compound and on the dissolution medium. One of the widely used equations to model the dissolution process is the Nernst-Brunner equation [28,29], reported below under the hypothesis that particles are spherical:


(7)

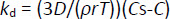



where *k*_d_ is the dissolution rate, *T* is the diffusion layer thickness, *C*s the solubility of the drug in the dissolving medium, *r*, *ρ* and *D* are the particle radius, density and diffusivity, respectively. The diffusion layer thickness can be computed following the approximation of Hintz and Johnson [30]:


(8)

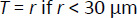



*T* = 30 μm, otherwise

The diffusivity can be calculated through the Stokes-Einstein equation:


(9)

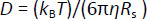



where *k*_B_ is the Boltzmann constant, *T* is the absolute temperature in Kelvin, *η* is the solute viscosity^1^ expressed in Pa·s, *R*_s_ is the hydrodynamic radius of the diffusing solute, calculated in (m), as [31]:


(10)

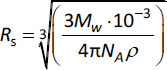



where *M*_W_ is the molecular weight, *N*_A_ is the Avogadro Number, *ρ* is the drug density (in kg·m^-1^).

Drug solubility is pH dependent except for non-ionizing compounds and it changes along the gut due to changes in the physiological pH. This can be taken into account by expressing solubilities at different pH values through the Henderson-Hasselbalch equations [32]. It depends on the specific nature of the compound (e.g. acid, base). An example is here reported for monoprotic acidic Eq. (11) and basic Eq. (12) compounds:


(11)

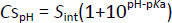




(12)

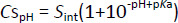



where *C*s_pH_ is the solubility at a certain pH; p*K*a is the acidic dissociation constant of the solute; *S*_int_ is the intrinsic solubility. For the other compound natures, relationships are reported in the Supplementary Material (*Compound-related parameter relationships* section).

### Absorption

The absorption rate constants of the gastrointestinal absorption model, *k*_a_, were calculated starting from the measurement of the apparent permeability, obtained from the *in vitro* Caco2 cell test. In particular from [33], *k*_a_ can be expressed as *P*_eff_2/*R*, where *P*_eff_ is the effective permeability and *R* is the intestinal radius. The value of human effective permeability, can be calculated following [34] as:


(13)

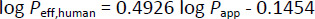



where *P*_app_ is the Caco2 apparent permeability measured at pH 7.4 and expressed in 10^-6^ cm/s. The resulting value of *P*_eff,human_ is expressed in 10^-4^ cm/s. Other relationships are present in [29] for PAMPA and MDCK assays. The effective permeability in rats, *P*_eff,rat_, can be obtained by the following relationship [34]:


(14)

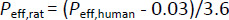



For dogs, in this work, it was assumed that *P*_eff,dog_ = *P*_eff,human_, because no specific information were found in the literature.

### Distribution

The compound-related parameters characterizing the drug distribution process are *P*_T:B_, defined as the ratio between the total drug concentration of a compound in a tissue T and the total drug blood concentration at the steady state. While in the past the values of these parameters originated from expensive and time-consuming experiments, nowadays they can be calculated by *in silico* models accounting for the tissue composition in terms of of water, lipids, and proteins. The two most widely used methods are the one developed by Poulin and co-workers [35,36] and the one proposed by Rodgers and co-workers, which extends the first one to take into account the nature of the compound (e.g., acid, base) and ionization phenomenon [37,38]. These models are reported for the reader in the Supplementary Material (*Compound-related parameter relationships* section).

### Metabolism and Elimination

The organs that generally constitute the major route of elimination in the organism are the liver and the gut, responsible for the first pass effect, and the kidneys.

Hepatic metabolic clearance can be obtained through the *in vitro - in vivo* extrapolation. It consists of three steps [39]: i) determination of *in vitro* intrinsic clearance *CL*_int_ (that can be obtained from hepatocytes, microsomes or recombinant enzymes); ii) scaling of *CL*_int_ to an *in vivo* value, here indicated as *CL*_int,viv__o_, by using appropriate scaling factor to obtain the value for the whole liver; iii) conversion of *CL*_int,vivo_ to a net value of hepatic clearance *CL*_H_. The scaling factor of step 2 is, in case of test with microsomes, the milligram of proteins per gram of liver (MPPGL) and, in case of hepatocytes, is the millions of hepatocytes per gram of liver (HPGL). Of course, these factors have to be multiplied for the liver weight to obtain the final value for the whole liver. The different values for rat, dog and man are reported in the Supplementary Material. One of the widely used models in step 3 to obtain *CL*_H_ is the well-stirred liver model [40], here reported:


(15)

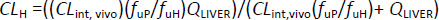



where *Q*_LIVER_ is the liver blood flow and *f*_u__H_ is the fraction unbound in microsomes or hepatocytes. If *f*_u__H_ is not known, it can be assumed that is equal to 1 (binding negligible) or that *f*_uH_ = *f*_u__P_ (the liver binding is equivalent to plasma protein binding) [7]. From *CL*_H_, the hepatic extraction ratio *E*_H_ can then be calculated as:


(16)

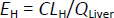



and then the resulting liver mass balance differential equation can be expressed as [32]:


(17)

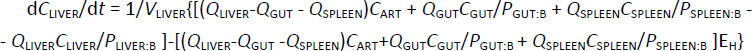



The intestinal contribution to the first pass metabolism can be obtained, in case of CYP metabolized drugs, by the “Qgut” model [34] as reported in the Supplementary Material. The equation describing the variation of the drug concentration in the gut can be expressed as follows [16]:


(18)

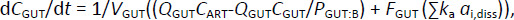



where *Q*_GUT_ and *V*_GUT_ are the gut blood flux and volume, respectively; *P*_GUT:B_ is the gut to blood partition coefficient; *F*_GUT_ is the fraction of drug that reaches gut escaping the intestinal first pass metabolism; *a*_i,diss_ is the amount of dissolved drug for the i-th compartment of the gastrointestinal absorption model.

The renal clearance can be calculated, in the simplest form neglecting processes of active secretion and tubular reabsorption, as GFR·*f*_u__B_ [25], where GFR is the glomerular filtration rate. The resulting equation for the kidney compartment is:


(19)

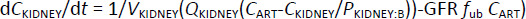



## Results and discussion

### Model evaluation

The PBPK model here presented was evaluated on IV and PO administrations by comparing model predictions with experimental data coming from the literature or in-house experiments, involving different drugs/compounds, administered in single dose or multiple doses, in three different species (i.e., rat, dog and man). Studies were selected to explore drugs with different physico-chemical properties and on the basis of the availability of all the drug parameters required to perform the simulations, summarized in Table 2.

The final list of selected compounds with the references in which experimental data and drug parameters were found is reported in Table 3.

Note that, as a general rule, all the PBPK model compound-related parameter values were computed, starting from the compound properties reported in Table 2, by applying the mathematical relationships reported in the previous sections and in the Supplementary Material. However, if the *in vitro* intrinsic clearance, required to characterize the hepatic metabolism, was not available, or the *in vivo* data of the plasma clearance *CL*_P_ was available, the hepatic extraction ratio *E*_H_ was directly calculated using the *in vivo* data as:


(20)

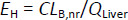



where *CL*_B,nr_ is the non-renal blood clearance obtained as *CL*_P,nr_/*BP* and *CL*_P,nr_ is the non-renal plasma clearance obtained after IV administration. If information about non-renal clearance were not available the total plasma clearance *CL*_P_ was used.

Moreover, *F*_GUT_ values in man were fixed to the values reported in the literature [53], if available, or calculated using the “Q_GUT_” model where possible; for the other species *F*_GUT_ was fixed to 1.

**Figure 3.**
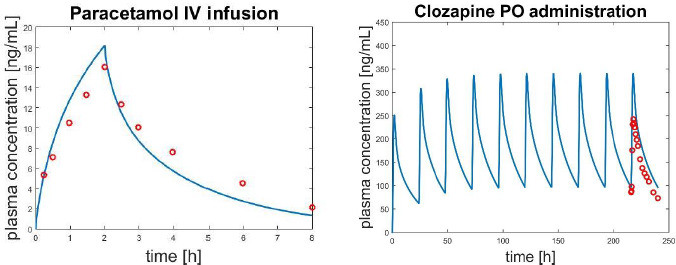
Predicted profiles (blue line) and the experimental data (red circles) in case of an IV infusion of Paracetamol (left panel) and a PO administration of Clozapine (right panel) in humans. For Paracetamol no adjustments were applied; for Clozapine, even if no IV data were available, the PS coefficient was increased to obtain a perfusion-limited kinetics.

Figure 3 reports, as an example, simulations related to an IV study (drug Paracetamol) and a PO study (drug Clozapine).

**Figure 4.**
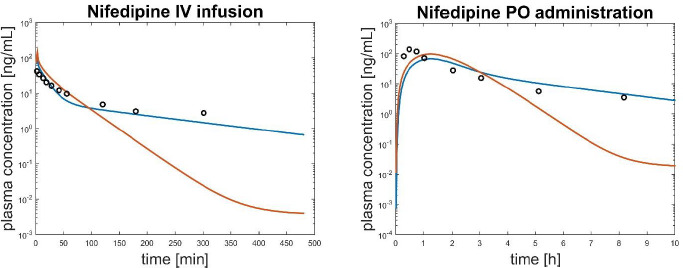
Comparison between PBPK simulated profiles tuning partition coefficients on IV data (blue line) and without tuning them (red line) for the drug Nifedipine. Experimental data are black circles. Left panel is related to an IV administration and right panel to PO administration.

When for the same drug both IV and PO data were available, PO predictions were obtained after having adjusted, if needed, model parameters on the IV data, as suggested by Peters in her work [16]. In particular, the drug distribution was adjusted multiplying all the partition coefficients for the same factor and tuning the PS parameters of the permeability limited tissues. In general, this procedure improved predictions of PO administrations. An example of the benefit of tuning the partition coefficients on IV profile is reported in Figure 4 for drug Nifedipine.

The choice of adjusting the distribution on the *in vivo* data and of using the *in vivo* clearance to calculate the hepatic extraction ratio wants to simulate the incremental model building process mentioned in the introduction, in which the knowledge is progressively added as soon as it is made available during the development process to refine the model. For highly lipophilic compounds, such as Clozapine and Amitriptyline, PS values were adjusted directly on PO simulations, when the IV data were not available, increasing PS values with the same factor^2^ to reach perfusion limited kinetics. This improved predictions when compared with real data.

Simulations were evaluated by considering the main pharmacokinetic parameters, i.e. area under the curve (AUC) of the plasma concentration-time profile, its maximum concentration values (*C*_max_) and the time to reach the maximum concentration (*T*_max_). In particular, AUC was considered for both the IV and PO experiments, whereas *C*_max_ and *T*_max_ were considered only for PO administrations and for IV infusions. The comparison between values computed on simulated and experimental profiles was made in terms of fold error:


(21)

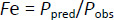



where *P*_pred_ is the PK parameter computed on the PBPK model predicted data and *P*_obs_ refers to those obtained from the experimental data.

**Figure 5.**
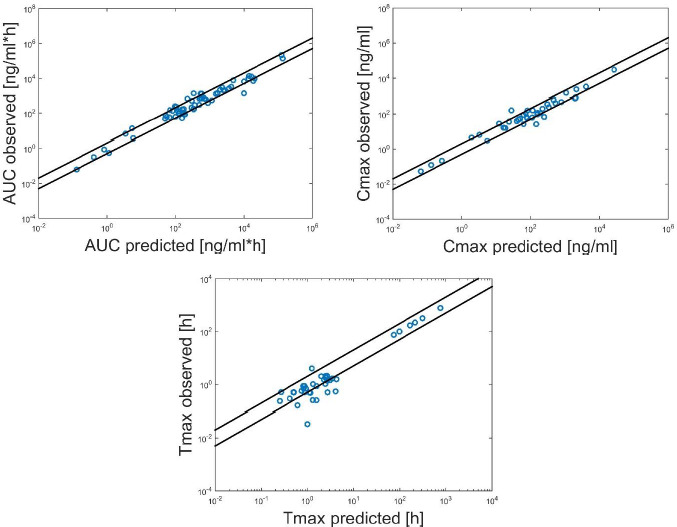
Comparison of the predicted and observed PK parameters, AUC in the upper left panel, Cmax in the upper right panel, Tmax in the panel below. The black lines represent the two-fold line deviation.

In Figure 5, the comparison between the predicted and observed parameters is shown.

It can be observed that for a large number of cases the predicted PK parameters are inside the two-fold limit.

Fold error values are reported in the Supplementary Material (*Model evaluation results* section) for all the compounds. As a metric of the overall performance the average fold error was calculated [16] for the three PK parameters as:


(22)

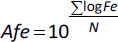



The average fold error for the AUC is 0.98, for the *C*_max_ 1.1 and for the *T*_max_ 1.37. The model showed good prediction performances since the Afe is for all the parameters under the two-fold limit. However, it should be considered that the use of the *in vivo* clearance data (where available) contributed to improve the predictive performances of the model, since is known that *in vitro* clearance measurements are often under predictive of the *in vivo* clearance [16]. Moreover, it should be noted that the PBPK model here presented does not consider possible kinetics caused by the action of specific enzymes and transporters that could play an important role in some cases. For example, they could allow the model to capture non-linearities in the PK due to some saturation effects. This behaviour can occur for mechanisms associated with absorption, first pass metabolism, binding, excretion and biotransformation; an example is the partial saturation of presystemic metabolism, one of the most important sources of non-linearity [75]. The modelling of specific kinetics could also be necessary to improve the model prediction capabilities in case of complex BCS class IV drugs, characterized by low solubility and low permeability for which the action of absorptive and efflux transporters could play an important role in their disposition [76].

## Conclusions

This paper shows, through a specific example, how it is possible to create in-house PBPK modelling tools, starting from the literature information and using some *in vitro* drug-specific properties that are typically routinely collected during the drug development process. Some references regarding how to model the basic ADME processes and where the main physiological parameter values can be found are also provided. For helping the reader, all the parameter values for rat, dog and man or the relationships to obtain these values are summarized in the Supplementary Material.

The developed PBPK model was evaluated in three different species on 25 compounds, intravenously and/or orally administered. It shows good performances in predicting the main PK parameters. The use of the *in vivo* IV data, when available, for the adjustment of the distribution parameters as well as for the prediction of the hepatic clearance proves to be a useful strategy to improve predictions of the plasma concentration-time profiles following other routes of administration.

This study demonstrates that it is possible to create simplified in-house PBPK tools, which can be the basis for the subsequent study of specific ADME mechanisms associated with special routes of administration. For example, our aim is to inform and support the drug development process by using the proposed PBPK model, extended with a pulmonary model we are working on, to study the PK of inhaled drugs. It is well known that a part of the inhaled drug can be swallowed immediately after inhalation or after the deposition for mucociliary clearance action. Hence, in this context, the possibility to correctly predict the gastrointestinal absorption can be useful to monitor the systemic exposure of inhaled drugs.

In this view, the evaluation of a PBPK model platform is an important and critical step of the model development, as also mentioned in both the EMA and FDA guidance [11,12]. In particular, the EMA guidance affirms that if a PBPK model is intended to support a regulatory decision, it has to be qualified for the intended use. Therefore, the evaluation of the model predictive performances and the level of qualification depend on the impact that the modelling exercise has on the decision making and on the patient’s risk associated to wrong regulatory decisions based on modelling predictions. Even if most of the regulatory submissions including PBPK models deals with the use of commercially available specialized (and validated) software tools [11], the use of in-house built platform is not discouraged by the regulatory agencies. In fact, FDA guideline does not prescribe the use of particular software for PBPK modelling and the EMA guideline applies also to in-house built PBPK platforms. However, it is clearly reported that if an in-house built platform is used for high regulatory impact simulations (e.g., as an alternative to clinical studies) the applicant is strongly encouraged to seek the Committee for Medicinal Product for Human Use (CHMP) Scientific Advice for further guidance [11].



## Figures and Tables

**Table 1:** - Values of the intestinal MRT for three species with references.

Species	Gastric MRT	Small intestine MRT	Colon MRT
Rat	Values reported in the literature show a certain variability, ranging from 2.7 min in [16] to 60 min in [26].	88 min [22]	228 min [25]
Dog	Values reported in the literature show a certain variability, ranging from 12.5 min in [16] to 96 min in [22].	109 min [22]	9.4 h, obtained by subtracting from the total gut transit time the stomach and the small intestine ones [22].
Man	Values reported in the literature show a certain variability, ranging from 15 min in [16] to 78 min in [22].	199.2 min [27]	11-92 h [25]

**Table 2:** - Drug specific information required to use the proposed PBPK model

Parameter	Symbol	Notes
**Nature of the compound**		e.g. acid, base, neutral, zwitterion
**Acidic dissociation constant**	p*K*a	e.g. obtained by *in silico* predictions
**Molecular weight**	MW	
**Particle Density**	*ρ*	Rarely available, a value frequently present in the literature is that used as default in the GastroPlus™ software [16,41,42], i.e. 1 *g/ml*.
**Particle radius**	*r*	If not available, a value suggested in the literature is that used as default in the GastroPlus™ software [43], i.e. 25 μm.
**Intrinsic solubility**	*C*_s_	If not available, but the solubility is known to a certain pH, Cs can be calculated with the Henderson-Hasselbalch equations.
***In vitro* apparent permeability**	*P*_app_	e.g. obtained from Caco2 or PAMPA test
***In vitro* intrinsic clearance**	*CL*_int_	e.g. obtained *in vitro* from microsomes or hepatocytes
**Blood to plasma ratio**	BP	e.g. obtained from *in vitro* test
**Fraction unbound in plasma**	*f*_uP_	e.g. obtained from *in vitro* test
**Log of n-octanol:water partition coefficient**	log *P*	e.g. obtained from *in vitro* test or *in silico* calculated
**Log of distribution coefficient (at a specified pH)**	log *D*_pH_	e.g. obtained from *in vitro* test or *in silico* calculated

**Table 3.** Compounds used for the evaluation of the proposed PBPK model with references in which the data and/or parameter values were taken from

Drug/Compound	Drug Parameters References	Study References
Amitriptyline	[44], [45], [46]	[45]
R-Carvedilol	[47]	[47]
Chlorpromazine	[48], [49], Drugbank, ChEMBL	[50]
Ciprofloxacin	[42], [51]	[52]
Clozapine	[48], [49], [53]	[54]
Compound A	Internal data	Internal data
Compound B	Internal data	Internal data
Compound C	Internal data	Internal data
Compound X	[3]	[3]
Digoxin	[55]	[56]
Diltiazem	[48], [49], [53]	[57]
Ibuprofen	[48], [49], [53]	[58]
Levothyroxine	[41], ChEMBL	[41]
Metoprolol	[59]	[59]
Midazolam	[19]	[60], [61]
Nifedipine	[62], [63]	[64], [65]
NVS732	[43]	[43]
Paracetamol	[66]	[66]
PF-02413873	[67]	[67]
Pracinostat	[68]	[68]
Repaglinide	[69], [70]	[71]
TPN729MA	[72]	[72]
Sotalol	[66]	[66]
UK-453,061	[73]	[73]
Verapamil	[48], [49], [53]	[74]

## Footnotes

^1^ In this work, the solute viscosity has been fixed to the water viscosity at 37 °C, i.e. 0.69 mPa s.

^2^ Amitryptiline, log *P* 4.85, *PS* correction coefficient 10^4^; Chlorpromazione, log *P* 5.18, correction coefficient 10^4^; Clozapine, log *P* 3.42, correction coefficient 10^3^; Verapamil, log *P* 4.05, correction coefficient 10^4^.
