# Supplementary material for: Building in-house PBPK modelling tools for oral drug administration from literature information
Source: ADMET DMPK. 2019 Feb 23;7(1):4–21. doi: 10.5599/admet.638 (PMC8957249; doi:10.5599/admet.638)
Supplement: Supplementary file 1 [file Admet-7-638-S1.pdf]

SUPPLEMENTARY MATERIAL

S. Grandoni et al. “Building in-house PBPK modelling tools for oral drug administration from literature information”

System-specific parameters values

Values of the physiological parameters used in the PBPK model described in the paper are here summarized. These values refer to a typical subject of 250 *g* for rats, 10 *kg* for dogs and 70 *kg* for man.

<u>*Rat parameters*</u>

**Fluxes [ml/min]**, [21,22]		**Volumes [ml],** [21,22,23]	
Q_{brain}	1.79	V_{brain}	1.43
Q_{gut}	11.92	V_{gut}	6.75
Q_{spleen}	0.8	V_{spleen}	0.5
Q_{liver}	14.6	V_{liver}	9.15
Q_{muscle}	24.91	V_{muscle}	101.8
$Q_{adipose}$	6.27	$V_{adipose}$	16.6
Q_{heart}	4.39	V_{heart}	0.83
Q_{kidney}	12.64	V_{kidney}	1.83
$Q_{Restofthebody}$	23.1	$V_{Restofthebody}$	72.36
Cardiac Output	89.6	V_{lung}	1.25
		V_{ven}	10.12
		V_{art}	3.38

**Gastrointestinal absorption model parameters**	
**Volumes of intestinal segments [ml]**, [25]	
$V_{stomach}$	3
V_1	0.6
V_2	0.66
V_3	0.66
V_4	0.41
V_5	0.41
V_6	0.41
V_7	0.41
V_{colon}	3
***pH* of intestinal segments**, [25]	
$pH_{stomach}$	3
pH_1	7.1
pH_2	7.3
pH_3	7.5
pH_4	7.7
pH_5	7.9

pH_6	8
pH_7	7.4
pH_{colon}	7.6
**MRT values**	
Stomach	10 min
Small intestine	88 min
Colon	228 min

Rat tissue composition to apply the Poulin's methods, [35]

Rat tissues	*Volume fraction of Phospholipids, Vph*	*Volume fraction of Neutral lipids, Vnl*	*Volume fraction of Water, Vw*	*Volume fraction of Interstitial space*
Adipose	0.002	0.853	0.12	0.715
Bone	0.0027	0.0273	0.446	0.42
Brain	0.0533	0.0392	0.788	0.162
Gut	0.0138	0.0292	0.749	0.39
Heart	0.0118	0.014	0.779	0.156
Kidney	0.0284	0.0123	0.771	0.346
Liver	0.0303	0.0138	0.705	0.159
Lung	0.014	0.0219	0.79	0.484
Muscle	0.009	0.01	0.756	0.115
Skin	0.018	0.0239	0.651	0.462
Spleen	0.0136	0.0077	0.771	0.264
Plasma	0.00083	0.00147	0.96	1
Erythrocytes	-	-	-	-

Rat tissue composition, to apply the method of Rodgers, [37]

Rat tissues	*Neutral phospholipids*	*Neutral lipids*	*Extracellular Water*	*Intracellular water*	*Tissue Concentration of Acidic Phospholipids (mg/g)*
Adipose	0.853	0.0016	0.135	0.017	0.40
Bone	0.017	0.0017	0.100	0.346	0.67
Brain	0.039	0.0015	0.162	0.620	0.40
Gut	0.038	0.0125	0.282	0.475	2.41
Heart	0.014	0.0111	0.320	0.475	2.25
Kidney	0.012	0.0242	0.273	0.483	5.03
Liver	0.014	0.0240	0.161	0.573	4.56
Lung	0.022	0.0128	0.336	0.446	3.91
Muscle	0.010	0.0072	0.118	0.630	1.53
Pancreas	0.041	0.0093	0.120	0.664	1.67
Skin	0.060	0.0044	0.382	0.291	1.32

Spleen	0.0077	0.0113	0.207	0.579	3.18
Thymus	0.017	0.0092	0.150	0.626	2.30

Tracheobronchial surface, S_{TB}: 81.75 cm^2 [14].

Hematocrit to compute the distribution: 0.46 [22].

Conversion factor to obtain the *in vivo* estimates of the hepatic clearance:

- MPPGL: 45 mg/g [25],
- HPGL: 125*10^6 cells/g [25],
- Liver Weight: 9.15 g [21].

Filtration parameter to model the renal clearance:

- GFR: 1.31 ml/min [22].

Dog parameters

**Fluxes [ml/min]**, [21,22]		**Volumes [ml],** [21,22,23]	
Q_{brain}	21	V_{brain}	78
Q_{gut}	216	V_{gut}	368
Q_{spleen}	24	V_{spleen}	27
Q_{liver}	288	V_{liver}	329
Q_{muscle}	227.9	V_{muscle}	456.5
$Q_{adipose}$	34	$V_{adipose}$	1380
Q_{heart}	48.3	V_{heart}	78
Q_{kidney}	181.65	V_{kidney}	55
$Q_{Restofthebody}$	246.8	$V_{Restofthebody}$	1538
Cardiac Output	21	V_{lung}	82
		V_{ven}	675
		V_{art}	225

**Gastrointestinal absorption model parameters**	
**Volumes of intestinal segments [ml]**, [25]	
$V_{stomach}$	14.54
V_1	30.54
V_2	32
V_3	32
V_4	20.1
V_5	20.1
V_6	20.1
V_7	20.1
V_{colon}	290.9
pH of intestinal segments, [25]	
$pH_{stomach}$	1.5
pH_1	6
pH_2	6
pH_3	6
pH_4	6.2
pH_5	6.2
pH_6	6.2
pH_7	7.4
pH_{colon}	6.5

**MRT values**	
Stomach	30 min
Small intestine	109 min
Colon	9.4 h

Tracheobronchial surface, S_{TB} =1176 cm^2, estimated with linear regression from the rat and man BW-S_{TB} data [14].

Haematocrit: 0.42 [22].

Conversion factor to obtain the *in vivo* estimates of the hepatic clearance:

- MPPGL 43 mg/g [25],
- HPGL 120*10^6 cells/g [25],
- Liver Weight 329 g [21].

Filtration parameter to model the renal clearance:

- GFR 61.3 ml/min [22].

Human parameters

**Fluxes [ml/min]**, [21,22]		**Volumes [ml],** [21,22,23]	
Q_{brain}	745	V_{brain}	1400
Q_{gut}	1046	V_{gut}	1155
Q_{spleen}	160	V_{spleen}	182
Q_{liver}	1578	V_{liver}	1799
Q_{muscle}	1055	V_{muscle}	28000
$Q_{adipose}$	310	$V_{adipose}$	14994
Q_{heart}	248	V_{heart}	329
Q_{kidney}	1179	V_{kidney}	308
$Q_{Restofthebody}$	1308	$V_{Restofthebody}$	10801
Cardiac Output	6204	V_{lung}	532
		V_{ven}	3900
		V_{art}	1300

Gastrointestinal absorption model parameters	
**Volumes of intestinal segments [ml]**, [25]	
$V_{stomach}$	50
V_1	105
V_2	110
V_3	110
V_4	69
V_5	69
V_6	69
V_7	69
V_{colon}	1000
pH of intestinal segments	
$pH_{stomach}$	2
pH_1	6
pH_2	6.2
pH_3	6.6
pH_4	6.8
pH_5	7
pH_6	7.2
pH_7	7.4
pH_{colon}	7

**MRT values**	
Stomach	30 min
Small intestine	199.2 min
Colon	11 h

Information available on human tissue composition, [35]

Human tissues	*Volume fraction of Phospholipids, Vph*	*Volume fraction of Neutral lipids, Vnl*	*Volume fraction of Water, Vw*
Adipose	0.002	0.79	0.18
Bone	0.0011	0.074	0.439
Brain	0.0565	0.051	0.77
Gut	0.0163	0.0487	0.718
Heart	0.0166	0.0115	0.758
Kidney	0.0162	0.0207	0.783
Liver	0.0252	0.0348	0.751
Lung	0.009	0.003	0.811
Muscle	0.0072	0.0238	0.76
Skin	0.0111	0.0284	0.718
Spleen	0.0198	0.0201	0.788
Plasma	0.00225	0.0035	0.945
Erythrocytes	-	-	-

Tracheobronchial surface, S_{TB}: 8990 cm^2 [14].

Haematocrit: 0.44 [22].

Conversion factor to obtain the *in vivo* estimates of the hepatic clearance:

- MGPPGL: 32 mg/g [S1],
- HPGL: 99*10^6 cells/g [S1],
- Liver Weight: 1799 g [21].

Filtration parameter to model the renal clearance:

- GFR 125 ml/min [22].

Drug-related parameters relationships

In this section the equations to calculate the drug-specific parameters are reported.

<u>Absorption</u>

The Henderson-Hasselbalch equations to calculate the solubility at a certain pH are here reported

Monoprotic acids

$C_{spH}=S_{int}(1+10^{(pH-pKa1)})$ (s1)

Monoprotic bases

$C_{spH}=S_{int}(1+10^{(-pH+pKa1)})$ (s2)

Diprotic acids

$C_{spH}=S_{int}\ (1+10^{(-pH+pKa1)}+10^{(2pH-pKa1-pKa2)})$ (s3)

Diprotic bases

$C_{spH}=S_{int}\ (1+10^{(-pH+pKa1)}+10^{(-2pH+pKa1+pKa2)})$ (s4)

Neutals

$C_{spH}=S_{int}$ (s5)

Zwitterions

$C_{spH}=S_{int}(1+10^{(-pH+pKaA)}+10^{(pH-pKaB)})$ (s6)

where pKaA is the acidic pKa and pKaB is the basic pKa.

<u>Partition coefficients</u>

This subsection contains the equations needed to calculate $P_{T:B}$ values with the method of Poulin [35,36] and of Rodgers [37,38]. For the latter the equations for each chemical species are reported.

Poulin's Method

The fractional volumes of phospholipids (Vph), neutral lipids (Vnl) and water (Vw), required to apply the method, are reported in the *species-specific parameters* section. In the following P indicates plasma and T tissue.

$Pow=10^{logP}$

$Dow=10^{logD}$

$f_{uT}=1/(1+(1-f_{uP})/f_{uP}0.5)$

For non-adipose tissues

$P_{T:p}=[(Pow(Vnl_T+0.3Vph_T)+(Vw_T+0.7Vph_T)]/[Pow(Vnl_p+0.3Vph_p)+(Vw_p+0.7Vph_p)](f_{up}/f_{ut})$ (s7)

For adipose tissues

$P_{T:p}=[(Dow(Vnl_T+0.3Vph_T)+(Vw_T+0.7Vph_T)]/[Dow(Vnl_p+0.3Vph_p)+(Vw_p+0.7Vph_p)]f_{up}$ (s8)

To obtain the values of $P_{T:B}$ from the $P_{T:P}$, the tissue to plasma partition coefficient, can be applied the following equation:

$P_{T:B}= P_{T:p}/BP$ (s9)

<u>Distribution</u>

Rodger's Method

The volumes related to tissues composition in terms of neutral lipids (nl), neutral phospholipids (nph), extracellular water (ew), intracellular water (iw), the ratios such as the lipoprotein ratio (lr), the albumin ratio (ar) and the tissue concentration of acidic phospholipids (ap) are reported in the *species-specific parameters* section. In the notation, T indicates the tissue and B the blood. The values of pHp, pHiw and pHbc are fixed, as reported by the authors, to 7.4, 7 and 7.22 respectively. The values for fNL_p and fNP_p are fixed as 0.0023 and 0.0013 respectively, as reported in the paper. For all tissues, except adipose ones, the value P in the subsequent equations is the n-octanol:water partition coefficient (here reported as P1); for the adipose tissues the vegetable oil:water partition coefficient was deemed more appropriate (here indicated as P2). To obtain the value of $P_{T:B}$ from the Kpu (tissue to plasma unbound partition coefficient) the following equation can be applied:

$$P_{T:B}=Kpu\ f_{uP}/BP \tag{s10}$$

$$P1=10^{logP} \tag{s11}$$

$$logPveg=1.115\ logP-1.35 \tag{s12}$$

$$P2=10^{logPveg} \tag{s13}$$

Acids

$X=1+10^{(pHiw-pKa)}$

$Y=1+10^{(pHp-pKa)}$

$$Kpu_T=ew_T+X\ iw_T/Y+((P\ nl_T+(0.3\ P+0.7)\ nph_T)/Y)+(1/fup-1-(P\ fNLp+(0.3\ P+0.7)\ fNPp)/Y)\ ar_T \tag{s14}$$

Diprotic acids

In this equations pKa1<pKa2

$X=1+10^{(pHiw-pKa1)}+10^{(-pKa2-pKa1+2pHiw)}$

$Y=1+10^{(pHp-pKa1)}+10^{(-pKa2-pKa1+2pHp)}$

$$Kpu_T= ew_T+X\ iw_T/Y +((P\ nl_T+(0.3\ P+0.7)\ nph_T)/Y)+(1/fup-1-(P\ fNLp+(0.3\ P+0.7)\ fNPp)/Y)\ ar_T \tag{s15}$$

Bases

$X=1+10^{(pKa-pHiw)}$

$Y=1+10^{(pKa-pHp)}$

$X1=1+10^{(pKa-pHbc)}$

$Y1=1+10^{(pKa-pHp)}$

$X2=10^{(pKa-pHbc)}$

$KpuBC=(BP-1+haematocrit)/haematocrit/f_{up}$

$KaAP=(KpuBC-(X1/Y1\ iw_b)-(P\ nl_b+(0.3\ P+0.7)\ nph_b)/Y1)\ (Y1/ap_b/X2)$

$$Kpu_T=ew_T+X\ iw_T/Y+(P\ nl_T+(0.3\ P+0.7)\ nph_T)/Y+(KaAP\ ap_T\ (X-1))/Y \tag{s16}$$

Very week bases

$X=1+10^{(pKa-pHiw)}$

$Y=1+10^{(pKa-pHp)}$

$$Kpu_T=ew_T+X\ iw_T/Y+((P\ nl_T+(0.3\ P+0.7)\ nph_T)/Y)+(1/fup-1-(P\ fNLp+(0.3\ P+0.7)\ fNPp)/Y)\ ar_T \tag{s17}$$

Diprotic bases

In these equations pKa1<pKa2

$X=1+10^{(pKa2\text{-}pHiw)}+10^{(pKa2+pKa1\text{-}2\ pHiw)}$

$Y=1+10^{(pKa2\text{-}pHp)}+10^{(pKa2+pKa1\text{-}2\ pHp)}$

$X1=1+10^{(pKa2\text{-}pHbc)}+10^{(pKa2+pKa1\text{-}2\ pHbc)}$

$X2=10^{(pKa2\text{-}pHbc)}+10^{(pKa2+pKa1\text{-}2\ pHbc)}$

$Y1=1+10^{(pKa2\text{-}pHp)}+10^{(pKa2+pKa1\text{-}2\ pHp)}$

KpuBC=(BP-1+haematocrit)/haematocrit/fup

$KaAP=(KpuBC\text{-}(X1/Y1\ iw_b)\text{-}((P\ nl_b+(0.3\ P+0.7)\ nph_b)/Y1))\ (Y1/ap_b/X2)$

$$Kpu_T=ew_T+X\ iw_T/Y+(P\ nl_T+(0.3\ P+0.7)\ nph_T)/Y+(KaAP\ ap_T\ (X\text{-}1))/Y \quad (s18)$$

Very week diprotic bases

In these equations pKa1<pKa2

$X=1+10^{(pKa2\text{-}pHiw)}+10^{(pKa2+pKa1\text{-}2\ pHiw)}$

$Y=1+10^{(pKa2\text{-}pHp)}+10^{(pKa2+pKa1\text{-}2\ pHp)}$

$$Kpu_T=ew_T+X\ iw_T/Y+((P\ nl_T+(0.3\ P+0.7)\ nph_T)/Y)+(1/fup\text{-}1\text{-}(P\ fNLp+(0.3\ P+0.7)\ fNPp)/Y)\ ar_T \quad (s19)$$

Neutrals

X=1

Y=1

$$Kpu_T=X\ iw_T/Y+ew_T+((P\ nl_T+(0.3\ P+0.7)\ nph_T)/Y)+(1/fup\text{-}1\text{-}(P\ fNLp+(0.3\ P+0.7)\ fNPp)/Y)\ lr_T \quad (s20)$$

Zwitterions, with at least one basic pKa>7

$X=1+10^{(pKaB\text{-}pHiw)}+10^{(pHiw\text{-}pKaA)}$

$Y=1+10^{(pKaB\text{-}pHp)}+10^{(pHp\text{-}pKaA)}$

$X1=1+10^{(pKaB\text{-}pHbc)}+10^{(pHbc\text{-}pKaA)}$

$Y1=1+10^{(pKaB\text{-}pHp)}+10^{(pHp\text{-}pKaA)}$

$X2=10^{(pKaB\text{-}pHbc)}+10^{(pHbc\text{-}pKaA)}$

KpuBC=(BP-1+haematocrit)/haematocrit/fup;

$KaAP=(KpuBC\text{-}(X1/Y1\ iw_b)\text{-}(P\ nl_b+(0.3\ P+0.7)\ nph_b)/Y1)\ (Y1/ap_b/X2)$

$$Kpu_T=ew_T+X\ iw_T/Y+(P\ nl_T+(0.3\ P+0.7)\ nph_T)/Y+((KaAP\ ap_T\ 10^{(pKaB\text{-}pHiw)})+10^{(pHiw\text{-}pKaA)})/Y \quad (s21)$$

All other zwitterions

$X=1+10^{(pKaB\text{-}pHiw)}+10^{(pHiw\text{-}pKaA)}$

$Y=1+10^{(pKaB\text{-}pHp)}+10^{(pHp\text{-}pKaA)}$

$$Kpu_T=ew_T+X\ iw_T/Y+((P\ nl_T+(0.3\ P+0.7)\ nph_T)/Y)+(1/fup\text{-}1\text{-}(P\ fNLp+(0.3\ P+0.7)\ fNPp)/Y)\ ar_T \quad (s22)$$

Metabolism and Elimination

The equations to apply the "Qgut" model [33], with the related scaling factors, to obtain F_{GUT} in humans from measurement of *in vitro* intrinsic clearance from HLM, for CYP3A metabolizers are here reported. The fraction of drug escaping the first pass metabolism can be calculated as follows:

$$F_{Gut}=Q_{villi}/(Q_{villi}+f_{uGUT}CL_{uint,GUT}(1+Q_{villi}/CL_{perm})) \quad (s23)$$

where Q_{villi} is the intestinal villi blood flow that for humans is 300 ml/min; f_{uGUT} is the unbounded drug fraction in gut, if not available can be supposed equal to 1; $CL_{uint,GUT}$ is the net metabolic intrinsic clearance based on the unbound drug concentration, this last term can be obtained from the HLM as follows:

$Cl_{uint,GUT}$ =(Cl_{uint}/PEMP)NEWI (s24)

where CL_{uint} is the unbound hepatic intrinsic clearance obtained from HLM and expressed in microliter/minute/milligram of protein, PEMP is the Picomol of CYP3A Enzymes for Milligram of Protein that is 155 picomol/milligram of protein, NEWI is the value of Nanomol of Enzyme for the Whole Intestine that is 70.5 nanomol [33].

The value of CL_{perm}, can be obtained as:

CL_{perm}=$Peff_{human}$ A (s25)

where A is the area of the intestine, for humans 6600 cm^2 obtained supposing a radius of 1.75 cm and a length of 6 m [33].

Additonal References

[s1] Zoe E. Barter, Martin K. Bayliss, Philip H. Beaune, Alan R. Boobis, David J. Carlile, Robert J. Edwards, J. Brian Houston, Brian G. Lake, John C. Lipscomb, Olavi R. Pelkonen, Geoffrey T. Tucker1 and Amin Rostami-Hodjegan. Scaling Factors for the Extrapolation of In Vivo Metabolic Drug Clearance From In Vitro Data: Reaching a Consensus on Values of Human Micro- somal Protein and Hepatocellularity Per Gram of Liver. *Current Drug Metabolism* **8** (2007) 33-45.
